# Correction: Huhtanen et al. Discrepancies between Radiology Specialists and Residents in Fracture Detection from Musculoskeletal Radiographs. Diagnostics 2023, *13*, 3207

**DOI:** 10.3390/diagnostics15010003

**Published:** 2024-12-24

**Authors:** Jarno T. Huhtanen, Mikko Nyman, Roberto Blanco Sequeiros, Seppo K. Koskinen, Tomi K. Pudas, Sami Kajander, Pekka Niemi, Eliisa Löyttyniemi, Hannu J. Aronen, Jussi Hirvonen

**Affiliations:** 1 Faculty of Health and Well-Being, Turku University of Applied Sciences, 20520 Turku, Finland; 2 Department of Radiology, University of Turku, 20014 Turku, Finland; sami.kajander@utu.fi (S.K.); pekka.niemi@utu.fi (P.N.); 3 Department of Radiology, Turku University Hospital, University of Turku, 20014 Turku, Finland; mikko.nyman@tyks.fi (M.N.); roberto.blanco@tyks.fi (R.B.S.); hannu.aronen@utu.fi (H.J.A.); jussi.hirvonen@utu.fi (J.H.); 4 Terveystalo Inc., Jaakonkatu 3, 00100 Helsinki, Finland; seppo.koskinen@terveystalo.com (S.K.K.); tomi.pudas@terveystalo.com (T.K.P.); 5 Department of Biostatistics, University of Turku, 20014 Turku, Finland; eliisa.loyttyniemi@utu.fi; 6 Department of Radiology, Faculty of Medicine and Health Technology, Tampere University Hospital, Tampere University, 33100 Tampere, Finland

Regarding our publication, entitled “Discrepancies between Radiology Specialists and Residents in Fracture Detection from Musculoskeletal Radiographs” [1], we have noticed errors in the original publication that require correction. The errors are mainly in the tables and text, where sensitivity and specificity values should be reversed. During the data collection, a normal (no fracture) radiograph was categorized as zero. In the data analysis phase, the SAS software version 9.4 for Windows (SAS Institute Inc., Cary, NC, USA) automatically assumed that the “disease” class was marked with a smaller number. Therefore, the SAS software calculated the sensitivity to the proportion of non-disease (no fracture) samples. This was not noticed during the writing process due to human error because the sensitivity and specificity numbers were very similar. These errors in the article do not have an impact on the scientific content of the study because they do not change the main results or conclusions made in the study. These errors have been corrected in Tables 2–4 and the article text accordingly. In addition, there was an incorrect number of overcalls, *n* = 31, which was corrected after we had examined the data. The amount of overcalls, *n* = 32, has since been corrected, and the relevant changes in the text (e.g., percentage changes) and Figure 1 have been made accordingly. In addition, typos due to human error have been corrected. The corrections made in the article do not change the main findings or conclusions in the study.

## 1. Corrections to the Number of Overcalls

In the original publication, there was an incorrect number of overcalls, *n* = 31, which was corrected after we had examined the data. The amount of overcalls, *n* = 32, has since been corrected, and the relevant changes in the text (e.g., percentage changes) have been made accordingly.

The CORRECTED paragraphs are as follows:

Abstract:

Of the 1006 radiographs, 41% were abnormal. In total, 67 radiographic findings were missed (6.7%) and 32 findings were overcalled (3.2%) in the original reports. The sensitivity, specificity, positive predictive value, and negative predictive value were 0.86, 0.92, 0.88, and 0.91, respectively.

3. Results, 3.1. Overall Findings, Paragraph 1:

Of the 1006 radiographs, 41% were abnormal. In total, 67 radiographic findings were missed (6.7%) and 32 findings were overcalled (3.2%). Among the missed fractures, 18% were found in children, 60% in adults, and 22% in elderly. Among the overcalls, 28.1% were found in children, 50% in adults, and 21.9% in elderly. The most common reason for interpretation error was fracture (58%). Interpretation error was most likely to happen in wrist (18%) or foot (17%) interpretations.

3. Results, 3.2. Discrepancies between Radiology Specialists and Residents, Paragraph 1:

No statistically significant differences (*p* = 0.44) were found in the interpretation errors between the radiology specialists and the residents. The radiology specialists missed 5.7% of the findings, while the residents missed 7.6%. On the other hand, the radiology specialists made 2.8% of the overcalls and the residents made 3.6% of the overcalls. The sensitivity, specificity, positive predictive value, and negative predictive value were 0.86, 0.92, 0.88, and 0.91, respectively (Table 3). Patient age was similar (*p* = 0.29) in the correct diagnosis group and in the interpretation error group. However, there were variations in competence between the different MSK regions and radiology specialists or residents.

Paragraph 4:

From all the missed findings in the radiographs, 70% (*n* = 44) were interpreted as having an impact on patient care (*p* = 0.02), but this did not differ between the radiology specialists and the residents. Findings missed by the radiology specialists (Figures 4 and 5) affected patient care in 71% of cases and overcalls in 31% of cases. Findings missed by the residents (Figure 6) affected patient care in 69% of cases and overcalls in 47% of cases. From all the overcalls in the radiographs, 40% (*n* = 12) seemed to have an impact on patient care. The most common impact on patient care was a lack of the necessary control study (40%), followed by an unnecessary control study (14%). Interpretation error rarely led to unnecessary operative treatment (1%).

4. Discussion, 4.2. Discrepancies between Radiology Specialists and Residents, Paragraph 3:

Diagnostic accuracy in the wrist had the lowest sensitivity and specificity among the MSK regions. This is worrying because the wrist is the most often injured MSK region [35,36], and missed findings can lead to complications such as nonunion, osteonecrosis, and osteoarthritis [6]. The radiology specialists and residents had the same miss rate, with 9.5% and 9.7%, respectively, but the radiology specialists had fewer overcalls compared to the residents, with 3.6% and 8.1%, respectively. These miss and overcall rates in the wrist are higher than reported in the previous studies [37]. Foot injuries are also very common, and diagnostic accuracy can have serious implications on patient care [38]. In our study, foot interpretation showed the lowest sensitivity and specificity in the lower extremity. These findings should prompt radiology departments to pay special attention to these MSK regions in resident training. We found that most interpretation errors affected patient care, regardless of whether the radiograph was interpreted by a radiology specialist or resident.

In the original publication, there was a mistake in Figure 1 as published. There was an incorrect number of overcalls, *n* = 31, which was corrected after we had examined the data. The amount of overcalls, *n* = 32, have been corrected, and the relevant changes in the text (e.g., percentage changes) and Figure 1 have been made accordingly. In addition, typos due to human error have been corrected. The corrected Figure 1 appears below.

## 2. Corrections to Sensitivity and Specificity Values

In the original publication, there was a mistake in Tables 2–4 as published. In Tables 2–4, the sensitivity and specificity values should be reversed. The corrected Table 2, Table 3 and Table 4 appear below.

**Table 2:** Diagnostic accuracy of radiology specialists’ and residents’ interpretations at different times.

	Sensitivity	Specificity	PPV	NPV
Daytime (8:00–16:00) (*n* = 444)	0.89 (0.85–0.94)	0.92 (0.89–0.95)	0.88 (0.84–0.93)	0.93 (0.90–0.96)
Daytime (8:00–16:00) Radiology specialist (*n* = 287)	0.89 (0.83–0.94)	0.92 (0.88–0.96)	0.88 (0.82–0.94)	0.92 (0.89–0.96)
Daytime (8:00–16:00) Radiology resident (*n* = 157)	0.91 (0.84–0.98)	0.92 (0.87–0.98)	0.90 (0.82–0.97)	0.93 (0.88–0.98)
Evening and night (16:01–07:59) (*n* = 562)	0.84 (0.79–0.88)	0.91 (0.88–0.94)	0.87 (0.83–0.91)	0.89 (0.85–0.92)
Evening and night (16:01–07:59) Radiology specialist (*n* = 219)	0.85 (0.77–0.92)	0.94 (0.90–0.98)	0.91 (0.84–0.97)	0.90 (0.84–0.95)
Evening and night (16:01–07:59) Radiology resident (*n* = 343)	0.83 (0.77–0.89)	0.90 (0.85–0.94)	0.85 (0.79–0.91)	0.88 (0.84–0.93)

PPV = positive predictive value, NPV = negative predictive value.

**Table 3:** Diagnostic accuracy of radiology specialists’ and residents’ trainee interpretations.

	Sensitivity	Specificity	PPV	NPV
UE and LE (*n* = 1006)	0.86 (0.83–0.90)	0.92 (0.89–0.94)	0.88 (0.84–0.91)	0.91 (0.88–0.93)
Radiology specialist (*n* = 506)	0.87 (0.82–0.91)	0.93 (0.90–0.96)	0.89 (0.85–0.93)	0.91 (0.88–0.94)
Radiology resident (*n* = 500)	0.86 (0.81–0.90)	0.90 (0.87–0.94)	0.86 (0.82–0.91)	0.90 (0.86–0.93)
UE (*n* = 495)	0.86 (0.81–0.90)	0.91 (0.87–0.94)	0.89 (0.84–0.93)	0.88 (0.85–0.92)
UE Radiology specialist (*n* = 249)	0.86 (0.80–0.93)	0.95 (0.91–0.99)	0.93 (0.88–0.98)	0.90 (0.85–0.95)
UE Radiology resident (*n* = 246)	0.85 (0.79–0.92)	0.86 (0.80–0.92)	0.85 (0.78–0.91)	0.87 (0.81–0.93)
LE (*n* = 511)	0.87 (0.82–0.92)	0.92 (0.89–0.95)	0.87 (0.82–0.92)	0.92 (0.89–0.95)
LE Radiology specialist (*n* = 257)	0.87 (0.80–0.94)	0.91 (0.86–0.95)	0.85 (0.77–0.92)	0.93 (0.88–0.97)
LE Radiology resident (*n* = 254)	0.86 (0.79–0.93)	0.94 (0.90–0.98)	0.89 (0.82–0.95)	0.92 (0.88–0.96)

PPV = positive predictive value, NPV = negative predictive value.

**Table 4:** Diagnostic accuracy of radiology specialists’ and residents’ interpretations at different MSK regions.

	Sensitivity	Specificity	PPV	NPV
Hand (*n* = 121)	0.82 (0.71–0.93)	0.94 (0.89–1.00)	0.91 (0.83–0.99)	0.88 (0.81–0.95)
Wrist (*n* = 125)	0.82 (0.73–0.91)	0.83 (0.73–0.92)	0.85 (0.76–0.93)	0.80 (0.70–0.90)
Elbow (*n* = 129)	0.92 (0.84–1.00)	0.94 (0.88–0.99)	0.90 (0.82–0.98)	0.95 (0.90–1.00)
Shoulder (*n* = 120)	0.88 (0.80–0.96)	0.90 (0.82–0.98)	0.90 (0.82–0.98)	0.89 (0.81–0.97)
Pelvis (*n* = 123)	0.97 (0.92–1.00)	0.95 (0.90–1.00)	0.95 (0.89–1.00)	0.97 (0.93–1.00)
Knee (*n* = 127)	0.88 (0.75–1.00)	0.92 (0.87–0.97)	0.73 (0.58–0.89)	0.97 (0.93–1.00)
Ankle (*n* = 136)	0.83 (0.72–0.93)	0.93 (0.87–0.98)	0.88 (0.78–0.97)	0.90 (0.83–0.96)
Foot (*n* = 125)	0.78 (0.67–0.90)	0.89 (0.82–0.96)	0.83 (0.73–0.94)	0.86 (0.78–0.94)

PPV = positive predictive value, NPV = negative predictive value.

The corrected paragraphs are as follows:

3. Results, 3.2. Discrepancies between Radiology Specialists and Residents, Paragraph 2:

Diagnostic accuracy in the different MSK regions showed a wide range of variation (Table 4). The highest sensitivity (0.97), specificity (0.95), negative predictive value (0.97), and positive predictive value (0.95) were found in the pelvis interpretation, while the lowest sensitivity (0.82), specificity (0.83), negative predictive value (0.80), and positive predictive value (0.85) were found in the wrist interpretation. Overall, the lowest sensitivity (0.78) was found in the foot interpretation. For the shoulder, the radiology specialists made the correct diagnoses in 95% of the cases, compared to 83% by the residents; for the knee, the radiology specialists made the correct diagnoses in 89% of the cases, compared to 97% by the residents. However, there were no statistically significant differences between the radiology specialists and the residents in the different MSK regions.

4. Discussion, 4.1. Overall Findings, Paragraph 1:

We found similar rates of misses and overcalls in the reading of the radiographs between the radiology specialists and the residents, with both groups having lower sensitivity compared to specificity, yet there were differences in competence among the different MSK regions. Neither day nor time of the day showed statistically significant differences in interpretation competence. These results highlight that there are no major differences between the radiology specialists and the residents in MSK radiograph interpretation. However, there are MSK regions that need more attention in the future regarding competence in radiograph interpretation. This will have direct implications for resident training programs. Importantly, there were no statistically significant group differences in the age distribution between the resident and specialist groups, suggesting that the main conclusions are not biased by age.

4. Discussion, 4.1. Overall Findings, Paragraph 2:

For the upper and lower extremities, we found a sensitivity of 0.86 and specificity of 0.92, which are lower than reported in the previous studies [16]. In contrast to the previous studies [16,17], we did not find any statistically significant increase in the radiology specialist or resident interpretation errors for the evening or night shifts compared to the daytime shifts. However, we did find that the residents, who can be more prone to fatigue-related errors [18,19], made more interpretation errors during the night shift compared to the morning or evening shift. The radiology specialists are also prone to fatigue-related problems in interpretation [17] and, in this study, we found that 18% of missed diagnoses occurred between 15:00 and 17:00, which highlights the fatigue-related errors in interpretation. Most missed diagnoses in this study were related to missed fractures, similar to the previous studies [20–22]. The prevalence of abnormality in our study was 41%, which is in line with prevalence in clinical practice [23] and does not overestimate the ability to detect abnormal cases [24].

4. Discussion, 4.2. Discrepancies between Radiology Specialists and Residents, Paragraph 2:

We did not find any statistically significant differences between the radiology specialists and the residents, which is in contrast to the previous studies, where the radiology specialists showed better diagnostic accuracy compared to the residents (*p* = 0.02) [32]. However, there are also studies showing no significant differences between the radiology specialists and the residents [1,20,25]. In addition, we did not find statistically significant differences in the interpretation of subtle or obvious radiology findings, in contrast to the previous studies [32]. In this study, the radiology specialists had higher rates of detection and higher diagnostic accuracy for subtle findings compared to the residents, which is consistent with the previous studies [18]. Because we excluded reports initially signed by both a trainee and a specialist (a signal of consultation), the potential bias from specialists affecting trainee reports is probably low. In addition, we did not find statistically significant differences between the radiology specialists and residents in different MSK regions, as in the previous studies [33]. In the previous studies [16,30], ankle interpretation showed the highest sensitivity (0.98) and specificity (0.95). In this study, the ankle sensitivity (0.83) and specificity (0.93) were lower. Furthermore, in this study, sensitivity was lower compared to specificity in all the MSK regions except the pelvis. This is well recognized in the field of radiology and can be related to litigation in missed findings [34].

## 3. Corrections to Calculation Errors

In the original publication [1], there was a mistake in Table 1 published. The percentages in Table 1 were calculated based on *n* = 506 evaluations and not *n* = 500 evaluations. These errors have been corrected. The corrected Table 1 appears below.

There was an error in the percentages of subtle findings, and these have been corrected. The percentages were taken from obvious findings due to human error.

**Table 1:** Patient demographics in different subsets.

Patient Demographics	Radiology Specialists’ Evaluation (*n* = 506)	Radiology Residents’ Evaluation (*n* = 500)	Total (N = 1006)
Age (y)			
Mean			45.4 (1–99)
1–16	88 (17.4%)	92 (18.4%)	11.6 (1–16)
17–64	255 (50.4%)	260 (52.0%)	36.7 (17–64)
>65	163 (32.2%)	148 (29.6%)	79.4 (65–99)
Sex			
Male	218 (43.1%)	226 (45.2%)	444 (44.1%)
Female	288 (56.9%)	274 (54.8%)	562 (55.9%)

The CORRECTED paragraph is as follows:

3. Results, 3.1. Overall Findings, Paragraph 2:

Different MSK regions had different rates of subtle and obvious radiographic findings (*p* = 0.001). Most subtle findings were found in the elbow (31%) and wrist (30%). Subtle radiographic findings occurred most often at 3 p.m.–4 p.m. (44%), 5 p.m.–6 p.m. (38%), and 9 p.m.–10 p.m. (38%).

The authors state that the scientific conclusions are unaffected. These correction have been approved by the Academic Editor. The original publication has also been updated.

## Figures and Tables

**Figure 1:**
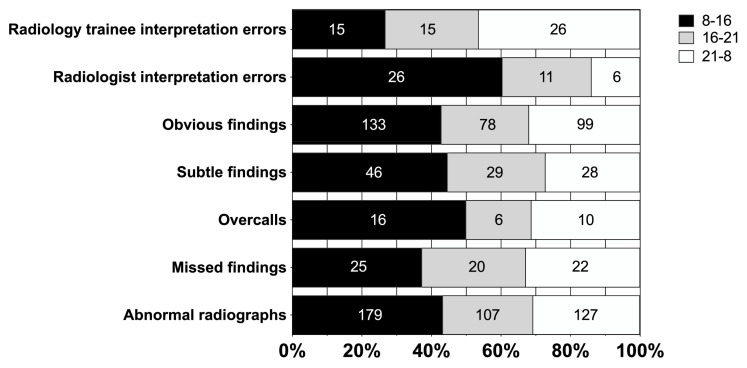
Total number and percentage of abnormal radiographs, missed diagnoses, and overcalls; subtle and obvious findings presented in three different timeframes.
